# Social observation differentially affects prosocial learning of selfish and prosocial people

**DOI:** 10.3389/fpsyg.2025.1440302

**Published:** 2025-02-12

**Authors:** Yuri Kim, Kun Il Kim, Hackjin Kim

**Affiliations:** Laboratory of Social and Decision Neuroscience, School of Psychology, Korea University, Seoul, Republic of Korea

**Keywords:** third-party observer, audience effect, reputation, reinforcement learning, prosociality, impression management, computational model

## Abstract

People often exhibit more socially favorable behaviors when observed by others, potentially influencing their cognitive skills and prosocial tendencies. Recent studies have found that individuals with intrinsic prosocial tendencies are non-responsive to social observation in various prosocial decision tasks. This study aimed to investigate whether individuals with intrinsic prosocial tendencies also exhibit a lack of change in their cognitive ability under social observation. We used the Prosocial Reinforcement Learning Task (PRLT) to assess the interaction effect of social observation and intrinsic prosocial tendency on prosocial learning tendency. A total of 102 participants were randomly assigned to either the observation or control group while performing a two-armed bandit task under self- and other-reward conditions, and their behavioral outcomes were analyzed using a reinforcement learning computational model. Under social observation, participants who were previously less prosocial exhibited increased prosocial learning. In contrast, those who were already more prosocial showed no significant changes in prosociality, and demonstrated only a numerical—but statistically non-significant—increase in learning for self. Our findings revealed the differential effects of social observation on modulating one’s prosociality and cognitive ability according to individual differences in intrinsic prosocial tendencies.

## 1 Introduction

People adapt to their environment through interactions with and influences from their surroundings. The impact of external factors on human behavior, along with personal factors such as personality traits that modulate these influences, has been a central topic in social psychology (Bolger and Zuckerman, 1995; Strombach et al., 2016; Chen et al., 2020; Ying et al., 2022; Saha et al., 2024). One key external factor is social observation, which refers to the awareness of being watched or evaluated. This phenomenon is a pervasive part of daily life, influencing decisions across various contexts—such as work, education, public policy, and personal social interactions.

Social observation motivates individuals to align their behavior with social expectations. For instance, employees may work harder to meet organizational goals, students may improve their academic performance, and consumers may choose ethical products to enhance their reputation. Zajonc (1965) introduced this phenomenon as the audience effect, where being observed motivates individuals to meet social norms or expectations. Even the mere presence or belief that one is being observed can elicit concerns about reputation, leading to impression management and socially desirable behavior (Andreoni and Bernheim, 2009; Milinski, 2016; Izuma, 2017; Jung et al., 2018; Yoon et al., 2018; Hofmeier and Strang, 2023; Liefooghe et al., 2023).

Existing studies have demonstrated that people tend to display prosocial behaviors under observation (Bradley et al., 2018; Cañigueral and Hamilton, 2019; Van Lange and Manesi, 2023). Social observation has also been shown to enhance cognitive abilities, such as memory and attentional control, particularly when individuals are able to and are motivated to perform better (Yantz and McCaffrey, 2005; McCaffrey et al., 2005; Sharma et al., 2010; Tian et al., 2015; Seymour et al., 2024; Garcia-Marques and Fernandes, 2024). Taken together, these findings indicate that individuals strive to be perceived as more socially desirable in response to social observation, depending on the aspects at stake in the context.

However, how individuals prioritize competing socially desirable attributes, such as competence and prosociality, under social observation remains unclear. Previous research has suggested that social observation might influence individuals differently depending on their social orientations, such as prosocial or self-centered tendencies (Simpson and Willer, 2008; Bereczkei et al., 2007; Bereczkei et al., 2010; Rotella et al., 2021; Li et al., 2023). This study addresses this gap by examining whether individuals prioritize cognitive performance or prosocial behavior in response to social observation, particularly depending on their intrinsic prosocial tendencies.

### 1.1 Social observation and behavioral change

Social observation, or the awareness of being observed, has been shown to influence individuals to act in socially desirable ways. Social observation has been studied at various levels, ranging from minimal interventions, such as presenting symbolic icons without direct explanation, to more explicit treatments that directly increase reputation concerns (Haley and Fessler, 2005; Barclay and Willer, 2007; Baillon et al., 2013; Worthy et al., 2024; Wang and Camerer, 2024). Although subtle differences exist across these levels, research has shown that even minimal treatment can significantly influence behavior (Van Lange and Manesi, 2023). For example, Haley and Fessler (2005) and Andreoni and Petrie (2004) demonstrated that observability increases charitable giving, suggesting a direct relationship between observation and prosocial behavior. Additionally, Lee et al. (2018) found that moral decisions tend to align more closely with social norms when individuals are under observation. Collectively, these findings highlight that people adapt their behavior to be perceived as socially favorable, cooperative, and generous.

Beyond prosocial behavior, social observation has also been shown to affect **cognitive performance**, often referred to as the social facilitation effect. For example, individuals demonstrate better memory recall and attentional control when being observed, particularly when tasks are within their capabilities (Yantz and McCaffrey, 2005; McCaffrey et al., 2005; Sharma et al., 2010; Tian et al., 2015; Seymour et al., 2024; Garcia-Marques and Fernandes, 2024). This facilitatory effect of social observation on cognitive performance becomes even more pronounced when environmental settings, such as online spaces, help reduce social anxiety that may arise from being observed (Strojny et al., 2020; Sutskova et al., 2023). These studies suggest that observation can enhance cognitive abilities, likely due to increased motivation to perform well in the presence of others.

### 1.2 Individual differences in response to social observation

Social orientations, such as prosocial or self-centered tendencies, may moderate how individuals respond to social observation. For example, individuals are more generous when their behavior is observed and information about their decisions is disclosed to others (Simpson and Willer, 2008). However, the effect of social observation might differ across groups: egoists are less prosocial in private conditions but show no significant difference in public settings compared to prosocial individuals (Rotella et al., 2021). These findings raise a critical question: Does the limited effect of social observation in prosocial individuals reflect low sensitivity to external factors, or is it due to the absence of alternative socially desirable values beyond prosociality? Addressing this issue requires examining whether individuals prioritize cognitive performance (competence) or prosocial behavior based on their intrinsic motivations.

### 1.3 Prosocial reinforcement learning task as a framework

To test these questions, we employed the Prosocial Reinforcement Learning Task (PRLT). This paradigm measures both learning abilities and prosocial tendencies by examining behavior under conditions in which individuals must learn from self-regarding and other-regarding outcomes (Sul et al., 2015; Lockwood et al., 2016; Lengersdorff et al., 2020; Westhoff et al., 2021). Prior studies using this task have revealed that individuals exhibit variations in learning patterns depending on their prosocial orientation. Specifically, prosocial individuals tend to learn more effectively from other-regarding rewards, whereas self-centered individuals prioritize learning from self-regarding rewards (Sul et al., 2015).

### 1.4 Research hypotheses

Based on the literature reviewed, we propose the following hypothesis: Individuals with greater intrinsic prosocial motivation may exhibit improved cognitive performance under social observation, as opposed to enhanced prosocial behavior. Alternatively, prosocial individuals may exhibit lower sensitivity to external social influences, as they may have already internalized prosocial values. Self-centered individuals are expected to show increased prosocial tendencies under social observation, particularly when learning for other-regarding rewards.

Using the task, where learning is intertwined with prosocial tendencies, we investigated whether participants employ diverse impression management strategies—either by demonstrating enhanced learning for themselves or by engaging in more prosocial value computation and examined whether the impact of social observation interacts with intrinsic prosocial tendencies. For individuals with more self-centered interests, we expected an increase in prosocial tendencies when they believed that they were being observed by a third-party, specifically showing an improvement in learning for other-regarding rewards as evidenced in previous studies. Importantly, we anticipated that individuals who previously considered prosocial value as important as the self-regarding values might display evidence of improved learning for self-regarding rewards to enhance “competent” attributes, an aspect less prioritized than “prosocial” attributes, or might not exhibit changes in learning for both self- and other-regarding rewards in response to social observation.

## 2 Materials and methods

### 2.1 Participants

We conducted *a priori* power analysis using the free-software G*Power 3.1 (Faul et al., 2007) based on the effect size of the interaction effect ( $\eta_{p}^{2}=0.103$ ), from a previous study that investigated observation on prosocial consumer decision-making (Jung et al., 2018). The power analysis suggested a sample size of 100 at *α* = 0.05 to achieve 95% power for 2 between- and 1 within-subject factors.

Participants were recruited via Korea University community website. All participants were fluent in Korean and had no prior experience with the reinforcement learning task. Inclusion criteria required participants to be between 18 and 30 years old, with no history of neurological or psychological disorders. A total of 112 participants (69 females, mean age = 23.5 years) were recruited and randomly assigned to either “Observation” (OBS) or “Control” (CON) group. Ten participants were excluded from the data analysis for the following reasons: one due to a technical issue, one due to misunderstanding the task instructions, and two due to missing more than half of the responses in a block. In addition, six participants who showed no evidence of learning—even for their own profit (i.e., whose learning rates for the “self” reward condition in the first block were estimated to be zero)—were excluded, as the learning rates in the first block were used to determine baseline learning tendencies. After applying exclusion criteria, 49 participants remained in the OBS group, and 53 participants remained in the CON group (Table 1). Following the experiment, participants were categorized as “selfish” or “prosocial” based on their prosocial tendencies during the control block (see parameter estimation and group categorization for details). The entire experimental procedure was approved by the Institutional Review Board of Korea University. Written informed consent was obtained from all participants before the behavioral experiment. After the experiment, all participants were debriefed about the deception and were compensated with KRW 8000 (approximately USD 6.7).

**Table 1:** Demographic characteristics of the observation and control groups.

Variable	Observation group (*n* = 49)	Control group (*n* = 53)	$X^{2}/t$	p
Gender (males/females)	17/33	21/32	0.002	0.963
Age (mean ± SD)	23.57 ± 2.40	23.49 ± 2.24	0.176	0.861
Age (min-max)	19–29	19–28		

### 2.2 Task design and procedure

#### 2.2.1 Stimuli

To minimize the effects of the subjective preferences for stimuli and similarity levels between each pair in the learning task, we carefully selected fractal image pairs as follows: from a previous study (Kim et al., 2021) where subjective preferences of a collection of 200 fractal images were rated by an independent group of participants, 16 fractal images with similar preference levels were selected for the behavioral task. We then generated 120 dyads from the 16 fractal images and another group of 47 participants evaluated the similarity of each pair using a 5-point Likert scale, ranging from 1 (not similar at all) to 5 (almost identical). For this study, we selected 6 pairs with similarity ratings between 1.5 and 2, which were subsequently used in the task (Supplementary Table S1).

#### 2.2.2 Experimental procedure

We conducted an online experiment using the metaverse platform “Gather.town”.^1^ Two participants were simultaneously invited to the online experimental space. All participants were instructed that the other participant would perform a different task while they completed three blocks of PRLT. Detailed information regarding the task assigned to the other participant was not provided. To prevent unwanted implicit influences, information about the social observation manipulation was withheld during the instruction period, because the first block was intended to measure baseline learning tendencies. To verify their understanding of the instructions, we asked several questions regarding the task rules. After providing correct answers, they could access online links to the behavioral task, which was programmed using psyToolKit (Stoet, 2010; Stoet, 2017).

**Figure 1:**
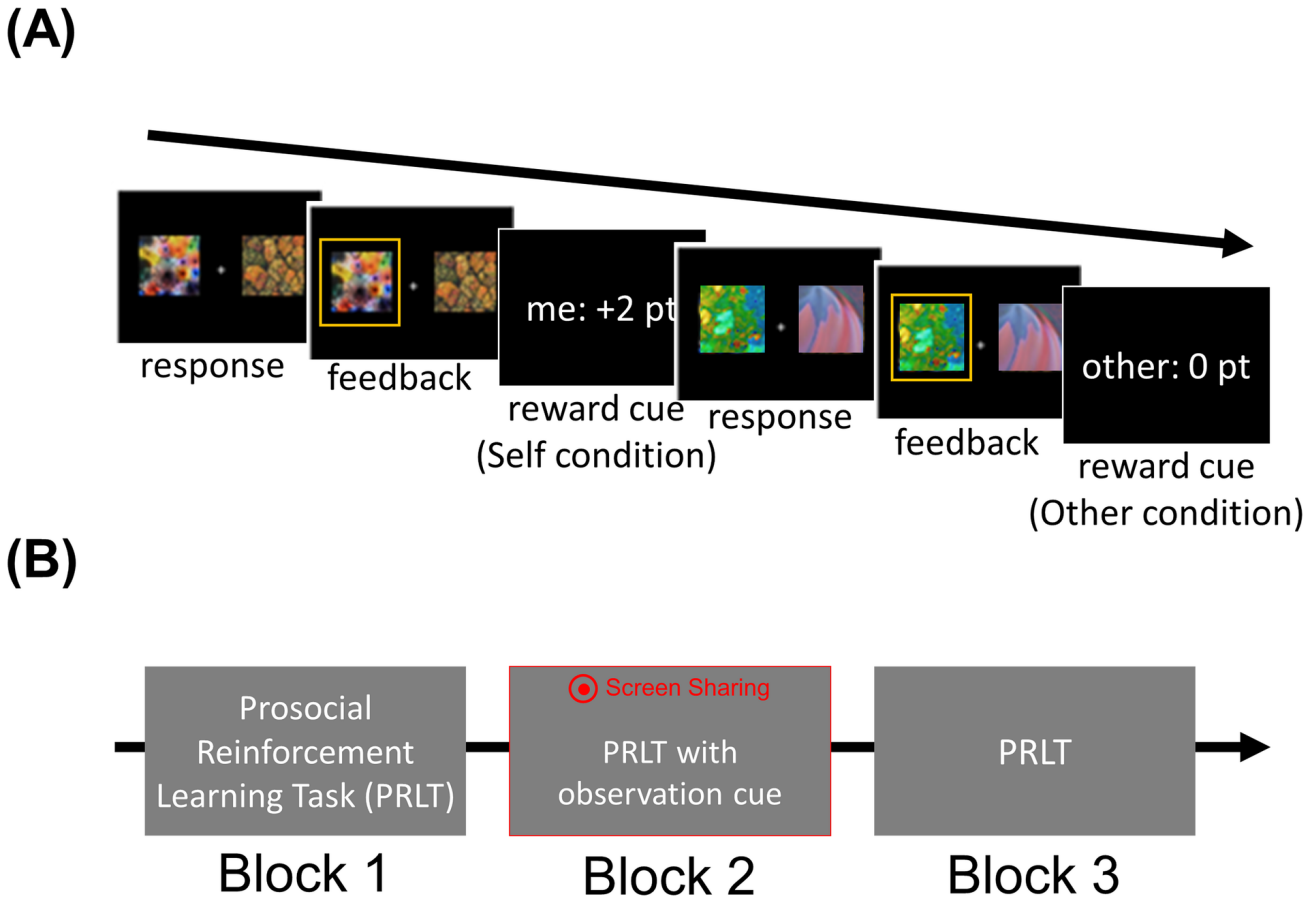
Prosocial reinforcement learning task (PRLT) and experimental procedure. **(A)** The sequential flow of two exemplary consecutive trials for the “self” and “other” reward conditions is shown. In each trial, a pair of fractal images was displayed until a response was made. Following the response, a feedback screen showing the chosen option was presented for 1 s. Subsequently, reward information, including the reward recipient and the reward outcome, was displayed after the feedback presentation. **(B)** Participants completed three blocks of the PRLT. During the second block, a red icon accompanied by the phrase “Screen Sharing” (in Korean) was displayed for participants in the “Observation’ group.” Before starting the second block, these participants were informed that their performance would be monitored by another participant. The third block was identical to the first block.

Before starting the main task, participants completed a training block to familiarize themselves with the task and were encouraged to ask any remaining questions regarding the task rules. Then, they performed three consecutive blocks of PRLT. Prior to the second block, the participants assigned to the OBS group were informed that the other participant would watch their performance via screen-sharing in real-time to utilize the performance information for another task. In addition, a red dot accompanied by the phrase “Screen sharing” displayed at the top of the screen to clearly indicate that their screen was being shared (Figure 1). This manipulation was designed to make the participants believe that their performance was being observed and implicitly evaluated by a third-party observer. After completing the second block, participants were informed that their performance would no longer be observed by the other participant.

After completing the main task, all participants were asked to guess the research hypothesis of the experiment. Then, they were debriefed about the deception that their counterpart participants had performed the same task as they did, and the participants in the OBS group were also informed that there had been no observation during the second block. None of the participants guessed the correct research hypothesis or refused to allow their data to be used.

#### 2.2.3 Prosocial reinforcement learning task (main task)

Within a single block, two pairs of fractal images were labeled as “self” (“self” reward condition) or “other” (“other” reward condition), respectively, indicating the target of the outcome associated with the pair. The two fractal images within a pair were associated with either a higher (70%) or lower (30%) probability of winning a point. The probability of winning for each image was reversed during the task. Participants would receive additional monetary incentives based on the points they earned in the “self” condition, as well as the points earned by the former anonymous participant on their behalf. Likewise, it was explained that the points they earned in the “other” reward condition would be given to the next participant. To maximize their own profit, participants needed to learn which pair was associated with self-regarding reward, and which option within the pair was associated with a higher reward probability.

The pair for “self” and “other” conditions was presented alternately. When one of the pairs was presented, participants were asked to choose between the two fractal images within 4 s or they would receive a message “You were late,” written in Korean. Participants were instructed that the recipient of the “other” reward would be the next participant in the following time slot (Figure 1). The chosen image was highlighted for 1 s. Subsequently, the outcome of the response was presented for 1.5 s.

### 2.3 Computational modeling

#### 2.3.1 Models for option valuation and value update

To measure individual differences in learning tendencies, we fitted individual responses to the reinforcement learning (RL) model (Sutton and Barto, 2018).

The softmax function was used to model trial-by-trial choice probabilities as follows:


$$P\left(t,a\right)=\frac{1}{1+e^{-\tau \left(V\left(t,a\right)-V\left(t,b\right)\right)}}$$

where *P*(*t*,*a*) is the probability of choosing option (fractal image) “*a*,” and *V*(*t*,*a*) or *V*(*t*,*b*) is the value of the option “*a*” or “*b*” in the trial *t*. According to the formula, the probability of choosing option “*a*” (*P*(*t*,*a*)) is based on the value difference of option “*a*” and “*b*” (*V*(*t*,*a*) – *V*(*t*,*b*)). The free parameter *τ* is the inverse temperature parameter indicating the sensitivity to the value difference. The choice probability of option “*b*” is calculated as 1−*P*(*t*,*a*).

According to the RL model, subjective values for each *V*(*t*,*a*) and *V*(*t*,*b*) are updated as follows:


$$V\left(t+1,a\right)=V\left(t,a\right)+\alpha \left(R\left(t\right)-V\left(t,a\right)\right)C\left(t,a\right)$$

$$V\left(t+1,b\right)=V\left(t,b\right)+\alpha \left(R\left(t\right)-V\left(t,b\right)\right)\left(1-C\left(t,a\right)\right)$$

$$C\left(t,a\right)=\left\{\begin{array}{l} \begin{array}{cc} 1, & \;{\mathrm{when option}}^{'}a^{\prime }\;\mathrm{was chosen} \end{array}\\ \begin{array}{cc} 0, & {\mathrm{when option}}^{'}a^{\prime }\;\mathrm{was not chosen} \end{array} \end{array}\right\}$$

where *R*(*t*) is the reward outcome of the trial, coded as 1 when the points were given and as 0 when the points were not given. The free parameter *α* is a learning rate, which represents how sensitively the value of the chosen option was updated from the prediction error (*R*(*t*) – *V*(*t*,*a*) or *R*(*t*) – *V*(*t*,*b*)).

#### 2.3.2 Model selection

Previous studies using similar prosocial learning paradigms reported differential learning for self- and other-regarding rewards (Sul et al., 2015; Lockwood et al., 2016; Lengersdorff et al., 2020). To ensure that it is reasonable to estimate separate learning rate and inverse temperature parameters, we compared models with shared (M1: α , τ ) and separate (M2: $\alpha_{\mathrm{self}}$ , $\alpha_{\mathrm{other}}$ , $\tau_{\mathrm{self}}$ , $\tau_{\mathrm{other}}$ ) free parameters for each reward condition by using Bayesian information criterion (BIC) scores. For fitting the RL model to behavioral data, we used the maximum likelihood estimation method. In addition, to ensure that the winning model appropriately explains the behavioral data, we compared the winning model with a null model in which the entire choice is assumed to be random, where the probability of choosing option “*a*” is always 0.5.

**Figure 2:**
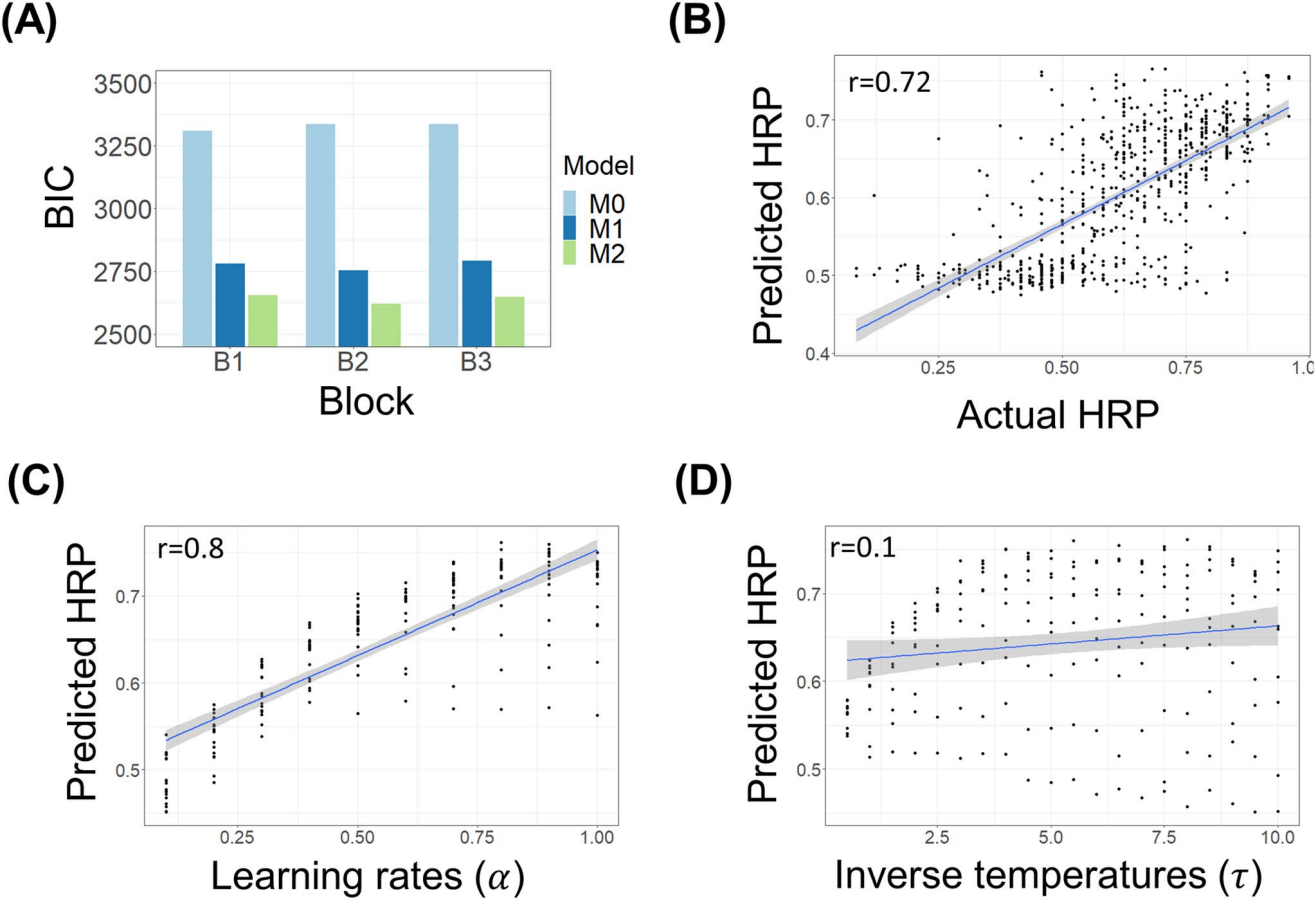
**(A)** Bayesian Information Criterion (BIC) scores comparing three models: the null model (M0), a model with shared free parameters across reward conditions (M1: α, τ ), and a model with distinct free parameters for each reward condition (M2: $\alpha_{\mathrm{self}}\text{,}$ $\alpha_{\mathrm{other}}\text{,}$ $\tau_{\mathrm{self}}\text{,}$ $\tau_{\mathrm{other}}$ ) across three blocks. **(B)** Posterior predictive check results comparing predicted High Reward Probability (HRP) with actual HRP. Predicted HRPs were generated using participant-specific free parameters, simulated 1,000 times, and averaged. The analysis provides qualitative evidence supporting the validity of parameter estimation. **(C)** Correlation between predicted HRP and learning rates $\left(\alpha \right)$ across varying inverse temperatures $\left(\tau \right)\text{.}$ **(D)** Correlation between predicted HRP and inverse temperatures $\left(\tau \right)$ across varying learning rates $\left(\alpha \right)\text{,}$ based on simulations of the winning model across combinations of *α*, ranging from 0.1 to 1, and *τ*, ranging from 0.5 to 10.

The model comparison results indicated that the model with separate learning rates and inverse temperatures for each reward condition (M2) was better at explaining the observed data compared to the alternative model (M1) in all three blocks (block1: ∆BIC = 125.5645; block2: ∆BIC = 133.7097; block3: ∆BIC = 143.0374; Figure 2A). Further comparison between the winning model and the null model (M0) revealed that the winning model better explains the trial-by-trial choices than the null model (block1: ∆BIC = 654.5; block2: ∆BIC = 714.97; block3: ∆BIC = 687.27). Using the parameters estimated from the winning model, we performed a posterior predictive check by generating predicted high reward probability (HRP) and found moderate to high congruence between the actual and predicted responses (*r* = 0.72; Figure 2B).

#### 2.3.3 Parameter estimation and group categorization

We generated predicted responses based on the winning model under every combination of α ranging from 0.1 to 1 and τ ranging from 0.5 to 10 and found that α was a better predictor of HRP compared to τ ( α:r=0.84 ; τ:r=0.14 ; Figures 2C,D). The simulation result was consistent with the previous report that the combination of high learning rates with moderately high inverse temperatures is reported as optimal for the highest choice accuracy under a learning environment with rapid reversals (e.g., every 10 trials) in an RL task (Zhang et al., 2020).

**Figure 3:**
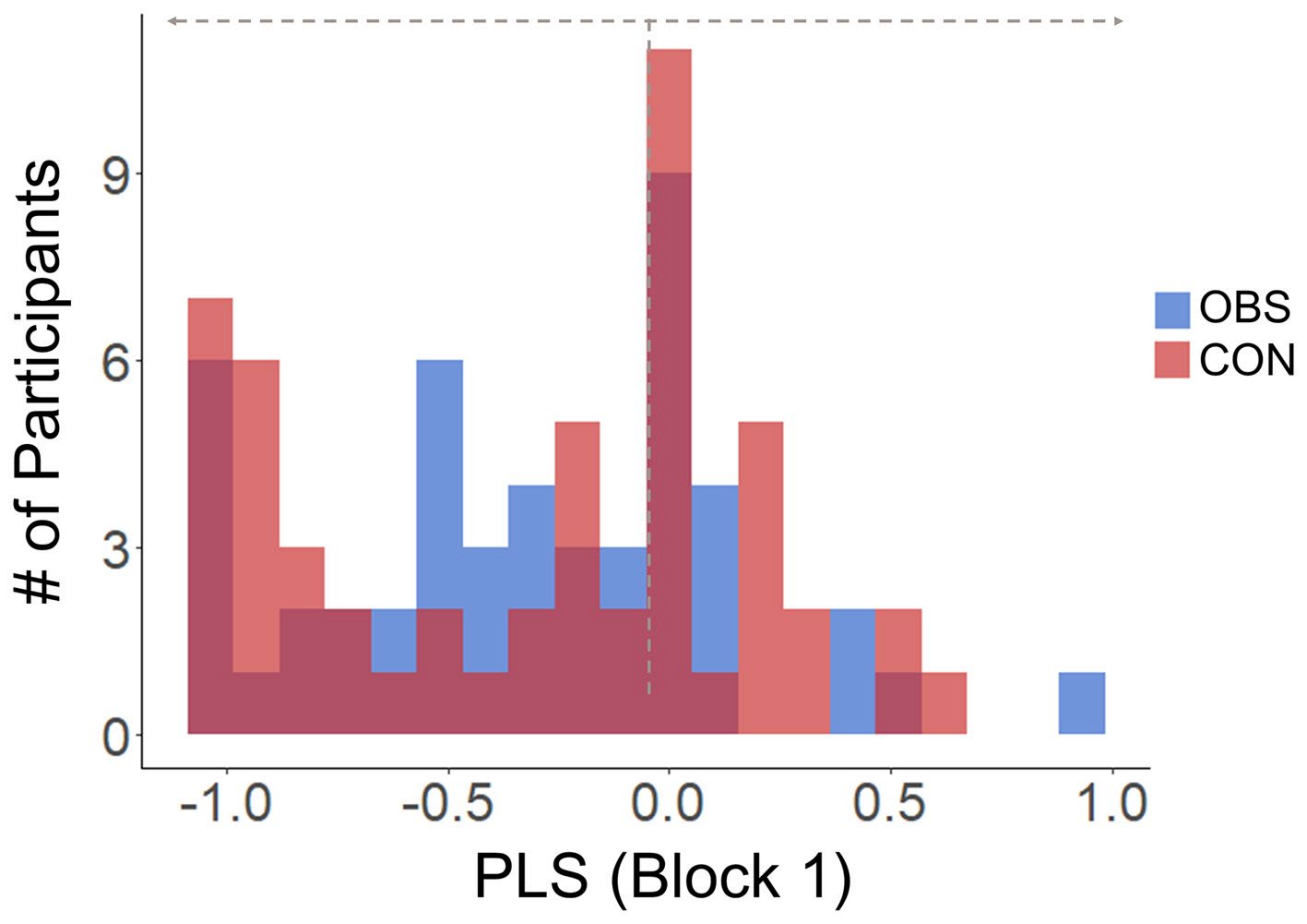
Distribution of prosocial learning sensitivities (PLS) in Block 1 for the Observation group (OBS; blue bars) and the Control group (CON; red bars) group. Participants with PLS values in Block 1 below 0 were classified as “Selfish,” while those with PLS values in Block 1 equal to or greater than 0 were classified as “Prosocial” group. The dotted vertical line marks the 0 value on the PLS scale.

As the learning rates ( α ) better represent learning tendencies than inverse temperatures ( τ ) in the current data, we computed participants’ prosocial learning sensitivities (PLS) by subtracting learning rates for “self” reward condition from “other” reward condition (PLS = $\alpha_{\mathrm{other}}-\alpha_{\mathrm{self}}$ ). Then we categorized participants into “Selfish” group if the PLS estimated from the first block ( $\mathrm{PL}S_{1}$ ) was smaller than 0, or “Prosocial” group if the $\mathrm{PL}S_{1}$ was equal to or larger than 0. Based on the individual PLS at the first block, 32 and 33 participants from OBS and CON group were categorized into the “Selfish” group, who showed higher learning rates in “self” compared to “other” condition, while the remaining 17 and 20 participants were categorized into the “Prosocial” group, who showed the same or higher learning rates for the other person compared to oneself, respectively (Figure 3).

### 2.4 Statistical analysis

The parameter estimation, model comparison, post-hoc pairwise *t*-test and data visualizations were performed using R version 4.2.0 (R Core Team, 2022) with “stats,” “rstatix” (Kassambara, 2021), and “ggplot2” (Wickham, 2016) packages. The *t*-test, one-way ANOVA for task manipulation check, and the four-way ANOVA for the behavioral analysis were performed using SPSS version 26.0 (IBM Corp., 2019).

## 3 Results

### 3.1 Task manipulation check

**Figure 4:**
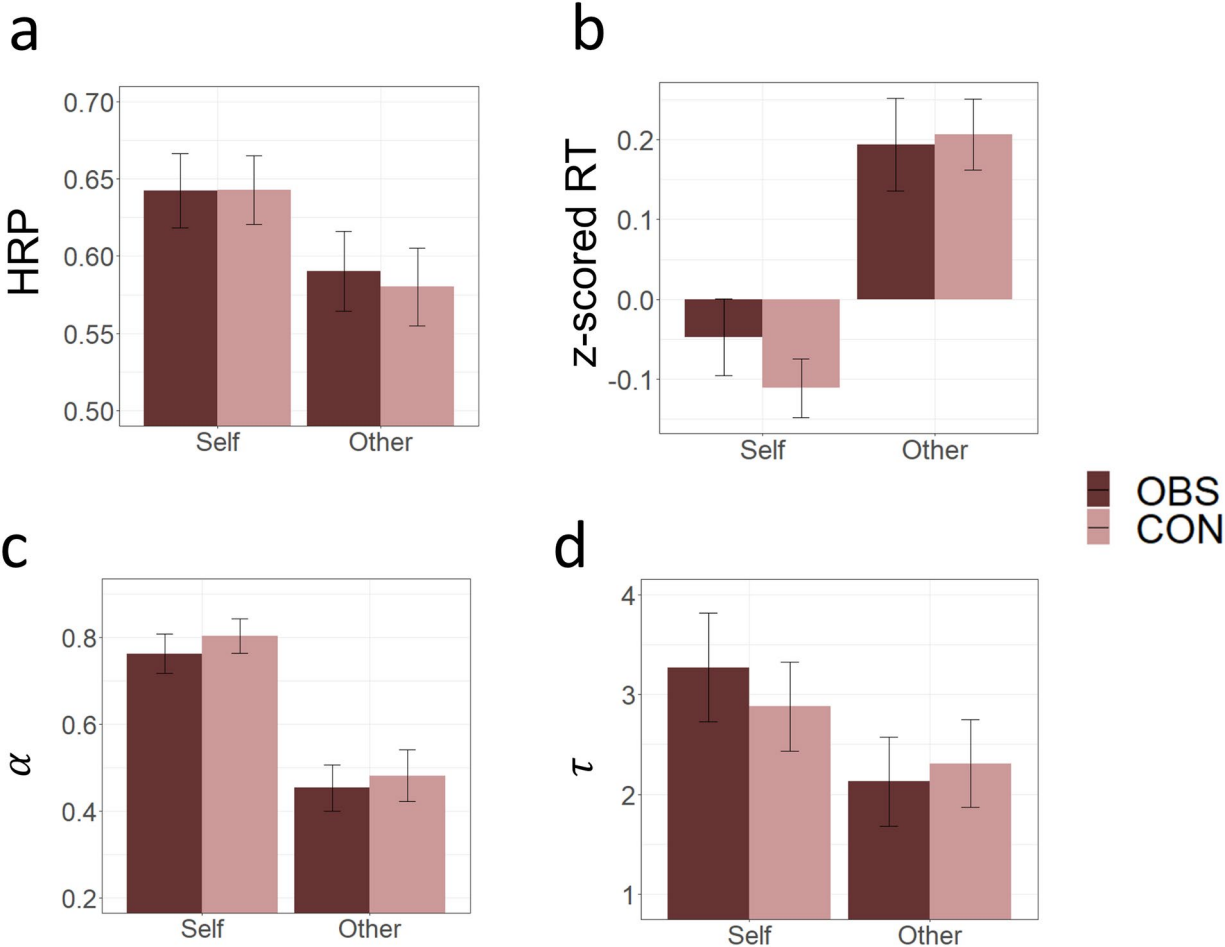
Comparison of task performance metrics between Observation (OBS) and Control (CON) groups in Block 1. **(A)** High Reward Probability (HRP), **(B)** z-scored Response Times (RT), **(C)** Learning rates $\left(\alpha \right)\text{,}$ and **(D)** Inverse temperatures $\left(\tau \right)$ for self- and other-regarding rewards. These comparisons serve as a task manipulation check to assess differences between the reward conditions and the groups.

We first examined whether participants successfully learned high reward probability (HRP) choice for “self” and “other” conditions at the first block. Participants chose HRP significantly above the chance level (Self: $t_{\left(101\right)}$ = 8.786; Other: $t_{\left(101\right)}$ = 4.731, both *p* < 0.001) and one sample *T*-test indicated that both learning rates (Self: $t_{\left(101\right)}$ = 26.458; Other: $t_{\left(101\right)}$ = 11.63, both *p* < 0.001) and inverse temperatures (Self: $t_{\left(101\right)}$ = 8.794; Other: $t_{\left(101\right)}$ = 7.102, both *p* < 0.001) were significantly higher than 0 for both “self” and “other” conditions. Comparisons between “self” and “other” reward conditions revealed differential learning for oneself and the other person. We observed significant target differences in HRP choice (Self vs. Other: $t_{\left(101\right)}$ = 2.894, *p* = 0.005), z-scored reaction time ( $t_{\left(101\right)}$ = 7.144, *p* < 0.001), learning rates, ( $t_{\left(101\right)}$ = 6.868*, p* < 0.001) and inverse temperatures ( $t_{\left(101\right)}$ = 1.991, *p* = 0.049), indicating that participants learned better and faster in “self” reward condition. To check if there was a group difference in baseline learning tendencies between the OBS and the CON groups, we conducted an independent samples *t*-test on hit rates, z-scored reaction time, learning rates, and inverse temperatures and did not find any group difference in the first block (all *p* values > 0.05; Figure 4).

We expected the PLS to correlate with behavioral variables and prosocial tendency survey scores. The correlation analysis revealed that PLS showed significant correlations with reward condition differences in HRP choice ( $HRP_{\mathrm{other}}-HRP_{\mathrm{self}};r_{\left(100\right)}$ = 0.31, *p* < 0.001), z-scored reaction time ( $RT_{\mathrm{self}}-RT_{\mathrm{other}}$ ; $r_{\left(100\right)}$ = −0.251, *p* = 0.011) in the first block. However, PLS in the first block showed only a marginal correlation with the social value orientation survey scores ( $r_{\left(100\right)}$ = 0.185, *p* = 0.063).

### 3.2 Effect of social observation

We hypothesized that social observation may evoke participants to either show better learning in the aspect of improvement in competence or show more prosocial value computation tendencies in the aspect of improvement in prosociality. We expected that the influence on competent aspect would result in increased learning rates for “self” reward condition, while the influence on prosocial aspect would result in increased learning rates for “other” reward condition or increased PLS.

To test whether there were changes in learning rates from the first block to second block specifically in the OBS group, we conducted 2 (Group: OBS, CON) × 3 (Block: 1, 2, 3) × 2 (Reward: self, other) factorial design repeated measures ANOVA on learning rates ( α) . However, the 2-way interaction effect (Group × Block; $F_{\left(2,\;200\right)}$ = 0.674, *p* = 0.511) and the 3-way interaction effect (Group × Block × Reward; $F_{\left(2,\;200\right)}$ = 1.119, *p* = 0.329) were not significant, indicating no evidence for the general effect of social observation.

### 3.3 Interaction effect of observation and PLS group

We further tested whether the lack of a general effect was caused by individual differences in impression management strategies. If individual differences in baseline PLS modulated the effect of social observation, the interaction effect of the PLS group would be significant. Therefore, we additionally conducted 2 (Observation: Observation, Control) × 2 (PLS group: Selfish, Prosocial) × 3 (Block: 1, 2, 3) × 2 (Reward: self, other) factorial design repeated measures ANOVA on learning rates ( α) .

**Figure 5:**
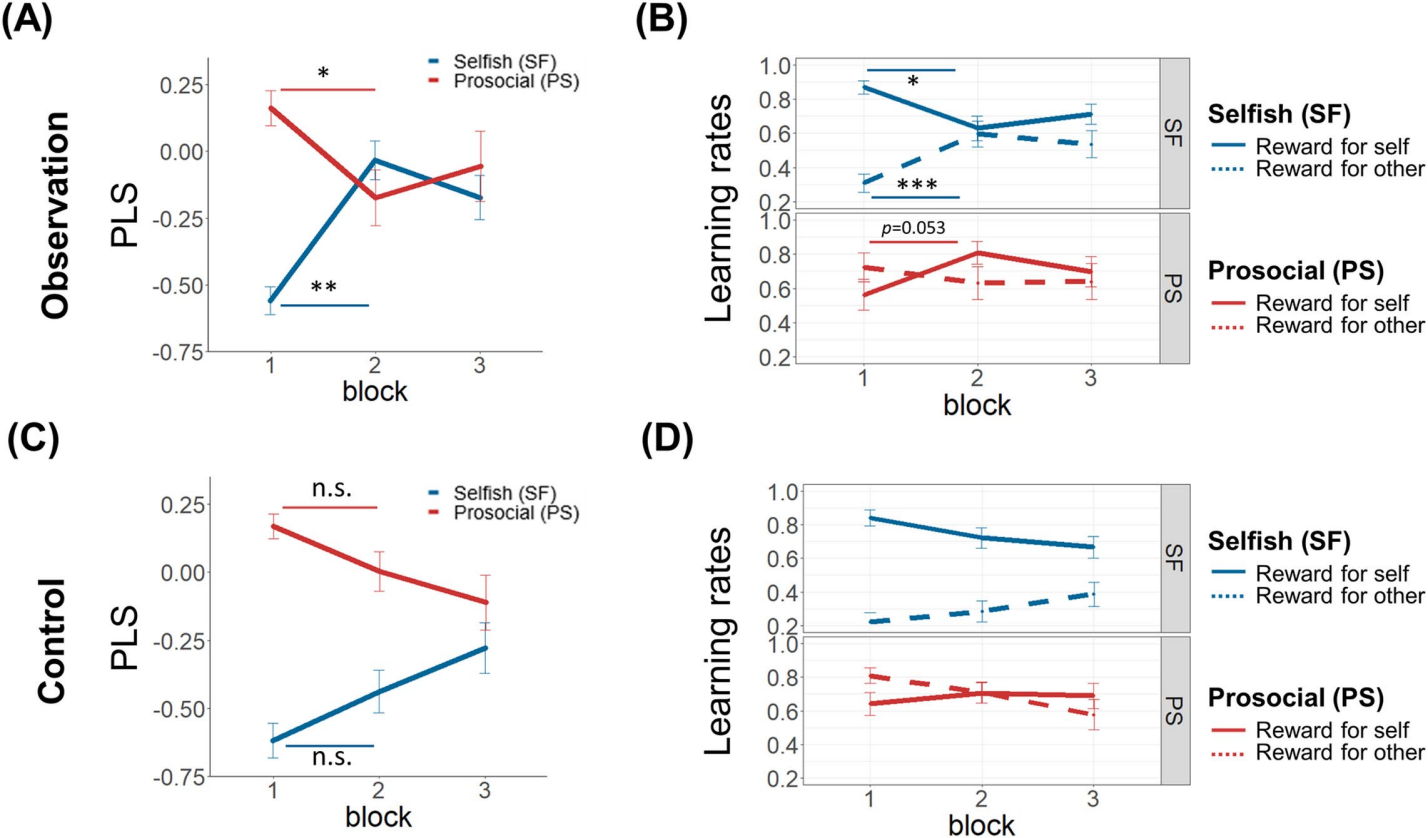
Results of the four-way interaction effect. **(A)** Changes in Prosocial Learning Sensitivities (PLS) across blocks for Selfish (SF; blue line) and Prosocial (PS; red line) groups within the Observation group. **(B)** Learning rates $\left(\alpha \right)$ across reward conditions, blocks, and group classifications (Selfish and Prosocial) within the Observation group. **(C)** Changes in PLS across blocks for Selfish (SF; blue line) and Prosocial (PS; red line) groups within the Control group. **(D)** Learning rates $\left(\alpha \right)$ across reward conditions, blocks, and group classifications (Selfish and Prosocial) within the Control group.

**Figure 6:**
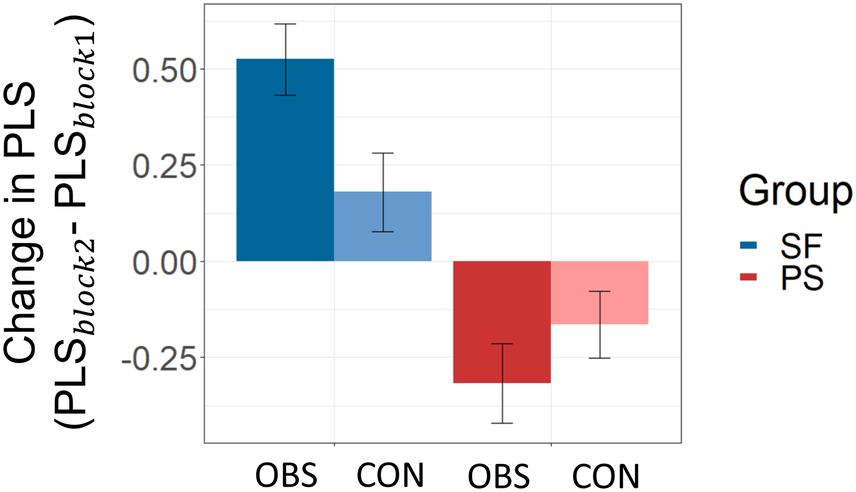
Results of the separate examination of changes in Prosocial Learning Sensitivities (PLS) across blocks for the Selfish (SF; blue) and the Prosocial (PS; red) group.

The four-way interaction effect between Observation, PLS group, Block, and Reward was significant ( $F_{\left(2,\;196\right)}$ = 3.616, *p* = 0.029; Figure 5). To ensure that the significant interaction effect was due to differential social observation effects in the PLS groups in accordance with our hypothesis, we conducted further post-hoc analyses with false discovery rate (FDR) corrections. The analysis revealed that the interaction effect was mainly due to the increased α for “other” ( $t_{\left(31\right)}$ = −4.918, *p* < 0.001, adjusted *p* < 0.001) and decreased α for “self” condition ( $t_{\left(31\right)}$ = 2.790, *p* = 0.009, adjusted *p* = 0.044) from first to second block in OBS-Selfish group (Figure 5B), while the same comparisons in the CON group was not significant (“self”: $t_{\left(32\right)}$ = 1.1614, *p* = 0.116; “other”: $t_{\left(32\right)}$ = −0.886, *p* = 0.382; Figure 5D). Further separate examination of PLS changes from the first to the second block between the OBS and CON group in the Selfish group revealed that the PLS change was significantly larger in the OBS group ( $t_{\left(63\right)}$ = 2.499, *p* = 0.015; Figure 6).

In OBS-Prosocial group, α for “self” condition marginally increased from the first to the second block ( $t_{\left(16\right)}$ = −2.532, *p* = 0.022, adjusted *p* = 0.053), while changes in α for “other” condition did not ( $t_{\left(16\right)}$ = 0.756, *p* = 0.461; Figure 5B). The same comparisons in CON group were not significant (“self”: $t_{\left(19\right)}$ = −0.707, *p* = 0.488; “other”: $t_{\left(19\right)}$ = 1.470, *p* = 0.158). However, further separate examination of changes in learning rates for “self” reward condition from the first to second block ( $\mathrm{Block}2\left[\alpha_{\mathrm{self}}\right]-\mathrm{Block}1\left[\alpha_{\mathrm{self}}\right]$ ) between OBS and CON groups did not show significant group difference ( $t_{\left(35\right)}$ = 1.338, *p* = 0.190). To assess the robustness of the results, a bootstrap resampling (*n* = 1,000) was performed for the same analysis on the changes in $\alpha_{\mathrm{self}}$ . The result confirmed that the finding was not due to the small sample size of the Prosocial group (*the bootstrap-corrected p* = 0.172).

There were no significant results for the HRP (probability of choosing options with high reward probability), reaction time, or inverse temperature parameters when performing the same analysis (Supplementary Figures S1–S3).

## 4 Discussion

In this study, we aimed to investigate whether the impact of social observation on social value computation varies based on inherent individual differences in social preferences. We delved into the nuances of how social observation operates in situations in which individuals must choose between distinct social values. We investigated alterations in reward sensitivity for self- and other-regarding learning under the influence of social observation, comparing these effects with those of a control group. Anticipating that the presence of a third-party observer would enhance prosocial behavior or overall learning, we hypothesized that the effect would differ among individuals based on their intrinsic prosocial tendencies. Our results revealed an interaction between social observation and individual differences in prosocial learning sensitivities, particularly evident in learning rates that reflect sensitivity to reward prediction errors.

Social observation had a differential impact on individuals with varying intrinsic prosocial learning sensitivities. It transformed previously selfish participants into more prosocial individuals, but did not significantly affect already prosocial participants. Consistent with studies on social observation and prosocial attributes, selfish participants exhibited increased prosocial learning sensitivity when observed by a third party, manifesting as a willingness to sacrifice personal gain for the benefit of an anonymous person. In contrast, prosocial participants did not exhibit significant differences between the observation and control groups, suggesting that external factors had limited influence on this group.

The limited impact of situational factors observed in previous studies and in the present study could be attributed to the notion that prosocial individuals may already process the values of prosocial behavior more automatically and intrinsically than their selfish counterparts. In line with the studies on situational factors, research on the effects of intranasal administration of oxytocin, which is known to accentuate emphasis on external social values in a context dependent manner, consistently demonstrated that the oxytocin effect was significant only in the selfish group, but not in the prosocial group, resulting in exclusive behavioral changes in the selfish cohort (Ma et al., 2016; Liu et al., 2019). Furthermore, a neuroimaging study utilizing a similar prosocial learning task revealed reversed patterns of spatial gradient in mPFC regions among selfish individuals, while prosocial individuals exhibited a shared neural representation of the self- and other-regarding value computation within the ventral medial prefrontal cortex, indicating that those with stronger prosocial learning tendencies intuitively process other-regarding values as self-regarding values (Sul et al., 2015).

Alternatively, the absence of a significant difference in the prosocial group may stem from the conflicting interests in maintaining a “prosocial” attribute. Although it was not statistically significant, there was a marginal increase in the learning rate for the “self” reward condition in the prosocial group when participants believed that they were being observed by a third-party. The decrease in the learning rate for “self” reward condition in the selfish group suggests that increasing learning sensitivity for one reward without sacrificing learning for the other is challenging in the current task set. The exclusive effect of social observation in selfish participants could indicate their willingness to sacrifice their own profit or the “competent” aspect of themselves for a more “prosocial” reputation. In contrast, prosocial individuals might choose not to sacrifice the other’s profit or “prosocial” aspect of themselves to maintain warm impression.

Taken together, these findings align with previous research, supporting the hypothesis that individuals prioritize prosociality over competence in the presence of social observation. Moreover, intrinsically prosocial individuals appear less responsive to social observation, likely because they have already internalized external values. These results suggest that theories of social facilitation should incorporate individual value systems as moderators. The present study also confirmed that social observation activates strategic prosocial behavior primarily in self-centered individuals, consistent with previous studies. This highlights the significance of how individuals prioritize warmth over competence in building their reputation across various social contexts. Furthermore, the effect of social observation is influenced by task design and stakes, underscoring the importance of integrating task complexity and observer characteristics into theoretical frameworks to better predict behavioral outcomes under observation. This nuanced understanding of the differential effects of social observation emphasizes the need for tailored approaches in industrial and educational settings to foster cooperation and competence among employees and students. Further research should explore whether third-party observer characteristics or task environments that emphasize specific attributes interact with intrinsic prosocial tendencies to modulate the effects of social observation.

Our study has several limitations that must be acknowledged. First, the differential social observation effect was solely evident in learning rates. Although previous studies have reported that inverse temperature parameters reveal differences between self- and other-regarding learning (Lockwood et al., 2016; Lengersdorff et al., 2020), our experimental setting did not show any relationship between inverse temperature and other learning indices or the difference between self- and other-regarding learnings. Although caution is advised when interpreting learning rate parameters in reinforcement learning paradigms in social psychological experiments (Zhang et al., 2020), we ensured that the learning rates were significantly correlated with other learning indices and marginally correlated with social value orientation survey scores. Second, the collection of cases for each selfish and prosocial learning tendency group was asymmetric. Almost 64% of the participants exhibited higher learning rates for the “self” than “other” reward conditions. This could limit the interpretation of the results of the prosocial group in our study, although we confirmed the robustness of the statistical findings through a bootstrap resampling.

In conclusion, under social observation, individuals with an intrinsically “selfish” learning sensitivity tended to enhance their “prosocial” aspect, partially sacrificing some of their own profit and impressions of competence, whereas those with an intrinsically “prosocial” learning sensitivity did not sacrifice other’s profit while enhancing the “competent” aspect of themselves, being less sensitive to situational factors, consistent with previous studies. These findings suggest that the effect of social observation interacts with intrinsic social preferences, and that social observation might facilitate social aspects that were previously considered less important for individuals, especially those lacking prosocial attributes.

## Data Availability

The original contributions presented in the study are included in the article/Supplementary material, further inquiries can be directed to the corresponding author.

## Footnotes

^1^ https://www.gather.town/
