# Supplementary material for: Social observation differentially affects prosocial learning of selfish and prosocial people
Source: Front Psychol. 2025 Feb 12;16:1440302. doi: 10.3389/fpsyg.2025.1440302 (PMC11860963; doi:10.3389/fpsyg.2025.1440302)
Supplement: Supplementary file 1 [file Data_Sheet_1.docx]

| Block | Reward condition | Image pair | Similarity |
| --- | --- | --- | --- |
| 1 | Self | 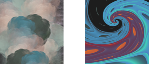 | 1.511  (0.906) |
|  | Other | 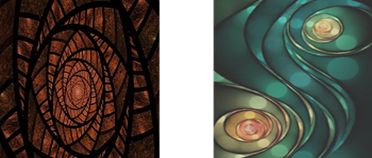 | 1.532  (0.997) |
| 2 | Self | 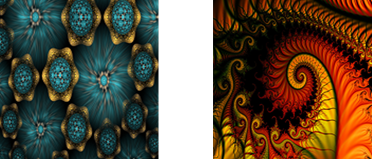 | 1.596  (1.077) |
|  | Other | 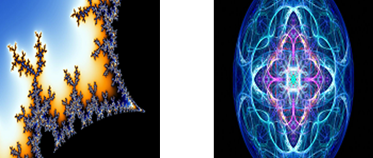 | 1.617  (1.153) |
| 3 | Self | 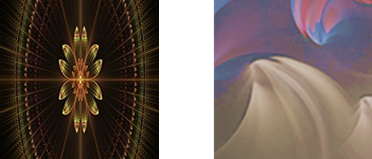 | 1.809  (1.056) |
|  | Other | 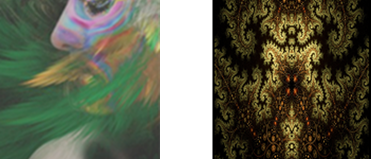 | 1.809  (1.076) |

**Table S1** The results of similarity ratings from an independent group of 47 participants. Participants rated the similarity between image pairs on a 5-point Likert scale, ranging from 1 (not similar at all) to 5 (almost identical). Six pairs with similarity ratings between 1.5 and 2 were selected and used in the task.


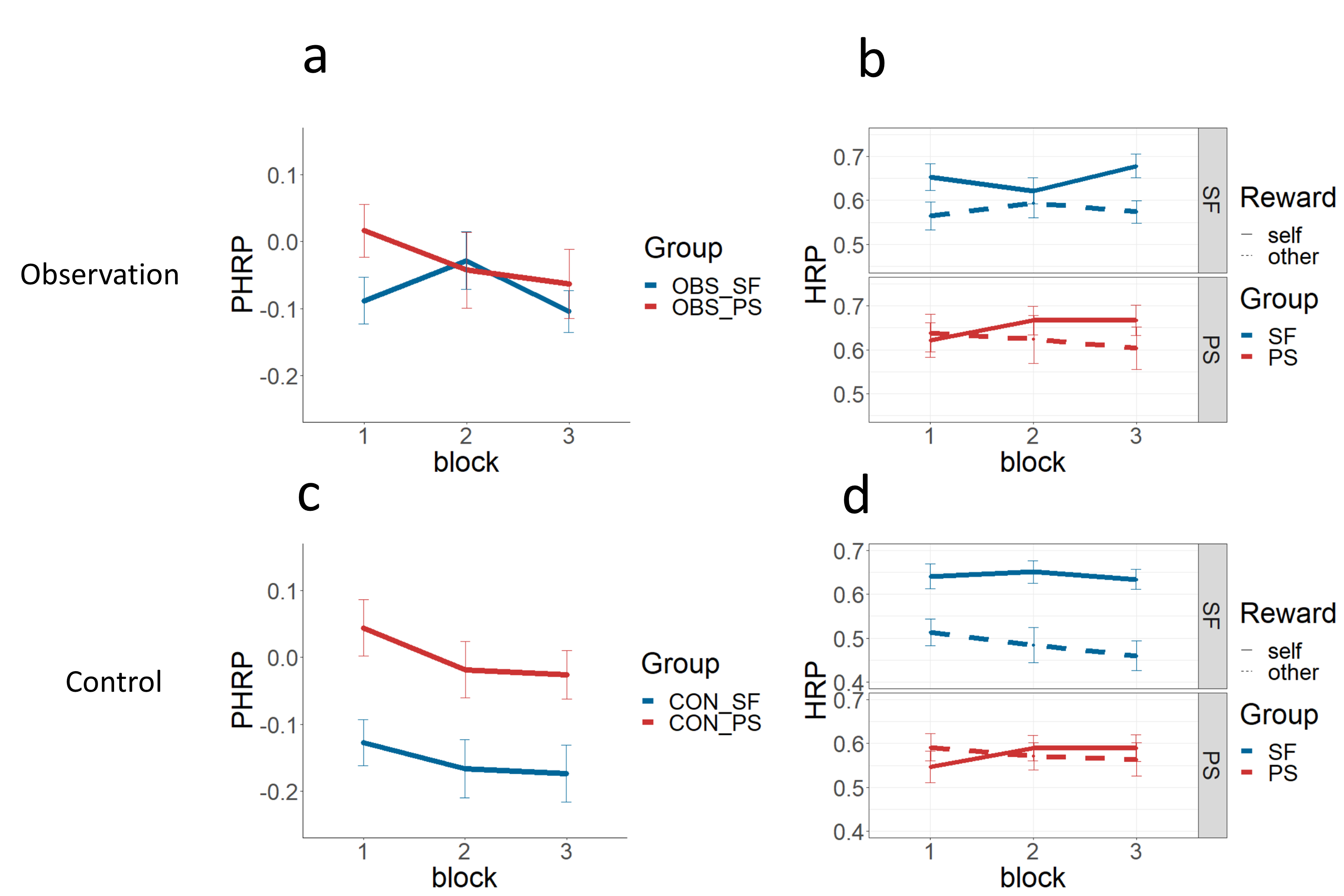


**Fig. S1** The results of the four-way interaction effect in HRP. (a), (c) Changes in HRP for each block in OBS and CON group. (b), (d) Learning rates for each reward condition, block and PLS group in OBS and CON group.


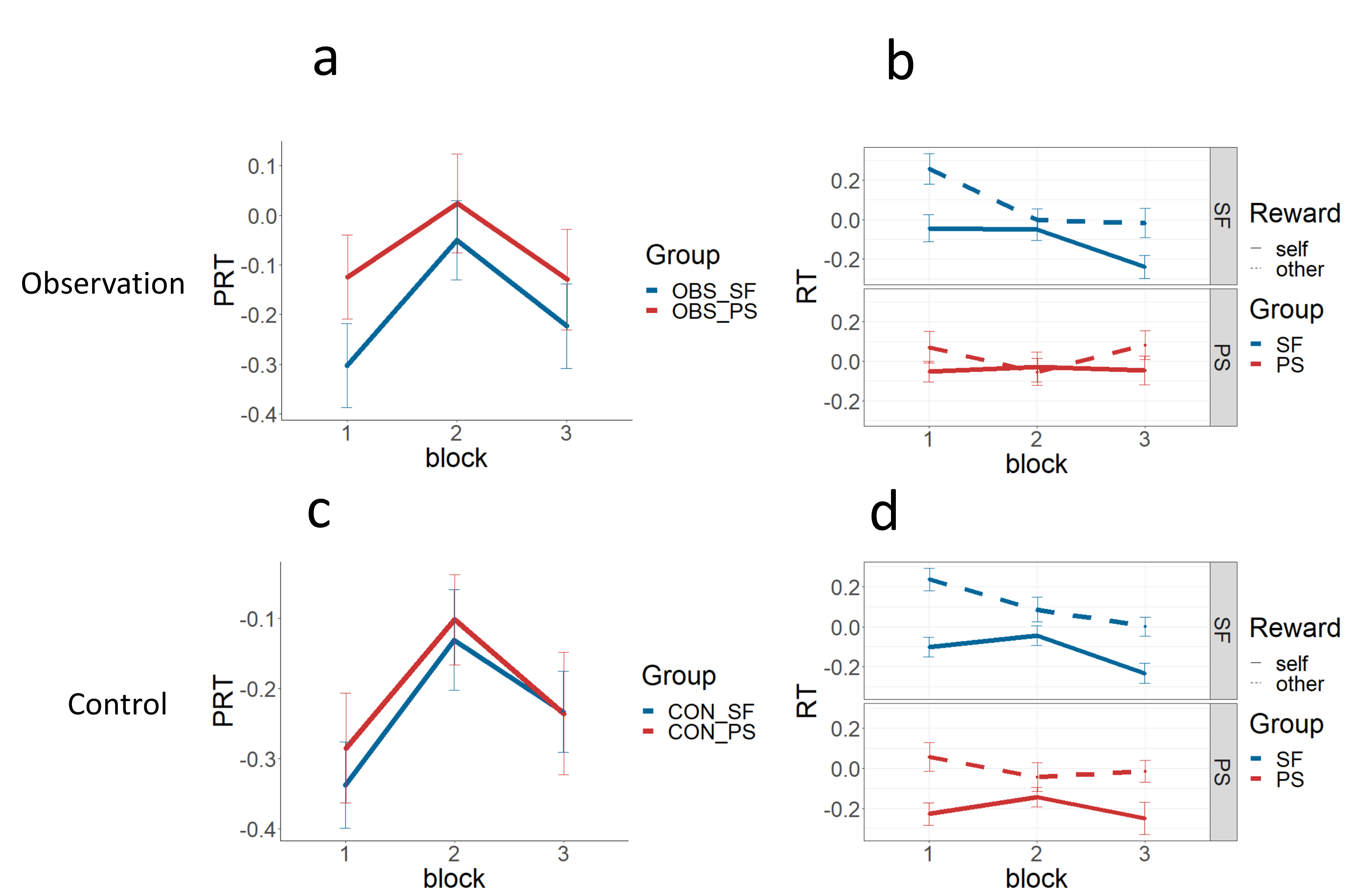


**Fig. S2** The results of the four-way interaction effect in reaction time (RT) and prosocial RT (PRT). PRT was defined by subtracting RT for ‘self’ condition from ‘other’ condition ($RT_{self}-RT\_other$), indicating how much it costs to choose an option for the ‘other’ reward condition than the ‘self’ condition. (a), (c) Changes in PRT for each block in OBS and CON group. (b), (d) RT for each reward condition, block and PLS group in OBS and CON group.

**
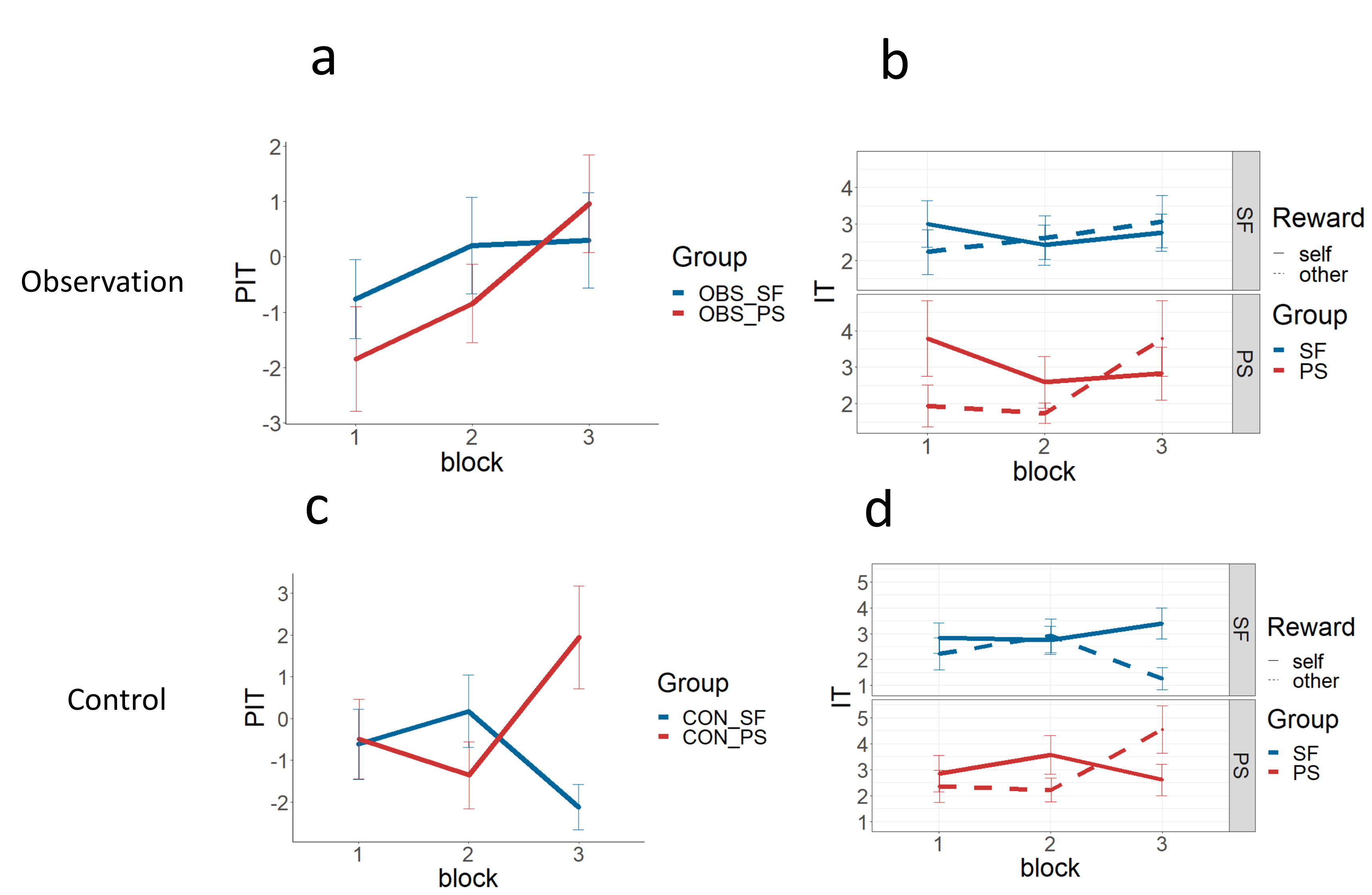
**

**Fig. S3** The results of the four-way interaction effect in inverse temperatures (IT) and prosocial IT (PIT). PIT was defined by subtracting IT for ‘other’ condition from ‘self’ condition ($IT_{other}-IT_{self}$). (a), (c) Changes in PIT for each block in OBS and CON group. (b), (d) IT for each reward condition, block and PLS group in OBS and CON group.
